# The Impact of the Australasian ‘Health Star Rating’, Front-of-Pack Nutritional Label, on Consumer Choice: A Longitudinal Study

**DOI:** 10.3390/nu10070906

**Published:** 2018-07-16

**Authors:** Robert Hamlin, Lisa McNeill

**Affiliations:** Department of Marketing, University of Otago, P.O. Box 56, Dunedin 9054, New Zealand; lisa.mcneill@otago.ac.nz

**Keywords:** front of pack, front of pack labelling, health star rating, nutrition labelling, food industry, traffic light label

## Abstract

Front-of-pack (FoP) nutrition labels are a widely deployed tool in public good marketing. This article reports on a field experimental test of the impact of one of these systems, the Australasian Health Star Rating system (HSR), on consumer choice in the breakfast cereals category in New Zealand. This study forms part of a time-series replication stream of research on this topic. The research applied a 2 × 2 factorial design with multiple replications to retail food consumers exiting from supermarkets in New Zealand. The first part of the time series, undertaken shortly after the HSR’s initiation in 2014, indicated that the HSR was ineffective. Between 2014 and 2016, commercial brands in the category within New Zealand massively promoted the HSR as a basis for consumer choice. The research presented in this article forms part of the second part of the series, undertaken in 2016, using an identical experimental methodology to the 2014 study. The results indicate that the HSR may be beginning to influence consumer choice as it was predicted to, but the impact of the system is still small, and statistically sub-significant, relative to other consumer decision inputs presented on the package.

## 1. Introduction

It is now generally accepted that over-consumption of certain nutrients, notably fat, sugar and salt, leads to increased morbidity and death [1]. Unfortunately, these ingredients are both cheap to produce and highly palatable, which has caused consumption of these nutrients and related undesirable health outcomes to increase, rather than decrease, in both developed and developing economies in recent decades [2].

The food industry also drives this increase in consumption by incorporating these ingredients into processed food products and marketing them to receptive segments of the population [3,4]. Sugar, which is reportedly addictive [5,6] and so cheap to produce that its commodity price is linked to that of petroleum [7], has started to attract particular attention in the last decade. Particularly, sugar has come under scrutiny with regard to the targeting of children with highly processed, value-added food products [8,9]. These matters attract researcher attention because the effects of this over-consumption not only lead to highly undesirable outcomes for the individual, but also lead to increased costs at the collective community level, especially for those states that deliver extensive state funded health interventions [10,11,12].

The response by states has developed progressively over the last 50 years [13]. Initially, efforts were based on the assumption that the consumer would respond to relevant nutritional information if it were offered [14]. Nearly all jurisdictions in developed countries now require the display of such information as a nutritional information panel (NIP) on food products [15]. However, a lack of measurable impact on consumer behavior that could be attributed to this ‘informed’ approach led to the development of a school of thought that an active persuasion, or ‘nudge’, might be required to change behavior [16]. This view has led to the development of front-of-pack (FoP) nutritional labels, which actively offer a third-party nutritional opinion/evaluation/interpretation to the consumer, at the point of decision and sale [17].

**Figure 1:**
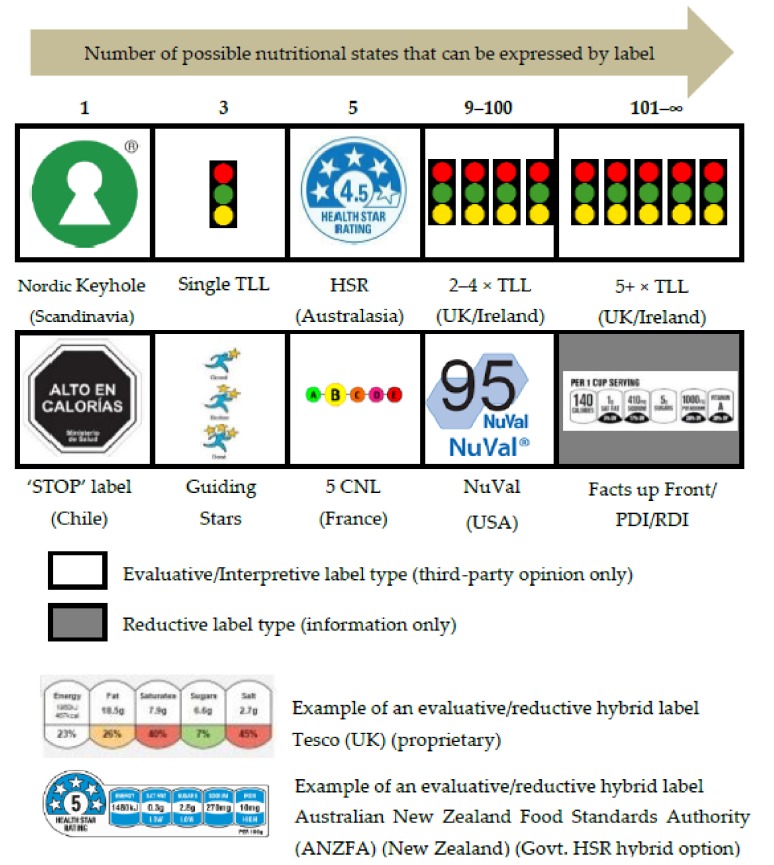
Major FoP nutritional labels types currently deployed around the World.

Over the last 20 years, a considerable amount of effort has gone into the development of ‘nudge’ initiatives in the form of FoP labels. Regrettably, this global developmental effort has not been coordinated, which has led to a situation where multiple FoP label formats, based on a wide variety of presentation and consumer processing mechanisms, have been deployed around the World (Figure 1) [18,19,20,21,22]. These different labels deliver different types of evaluative (nudge) content, ranging from 100% evaluative, which offers only an opinion to the consumer (e.g., ‘Nordic Keyhole’) to 100% reductive/informative which offers only information, without opinion (e.g., ‘Facts up Front’) [22]. A range of hybrid types that offer both information and opinion via a combination of separate evaluative/interpretive and reductive systems have also been employed (Figure 1).

In recent years, fully evaluative nutritional labelling systems have begun to predominate and proliferate, with the fully evaluative Chilean ‘STOP’ and French 5-CNL systems being the most recent major additions to the FoP portfolio [23,24]. The majority of these FoP labels are complex, with multiple states, and do not conform to the established marketing practice of single state (nominal) brand cues, that the food industry has historically used to effectively influence consumer choice [22]. This fragmented situation both reflects, and is aggravated by, a relative deficiency in published research that directly tests the impact of any of these FoP label types on unprompted consumer choice between food products. As a result, it is still not known for sure if any of these labels fulfil their purpose by significantly influencing unprompted consumer choice at the point of sale. The editorials of two major special issues of leading international journals have addressed this deficiency directly [17,25].

Since the appearance of Lachat & Tseng’s (2013) [25] editorial addressing the lack of direct testing of FoP labels, there has been a marked increase in the number of published research that tests their performance. This research output has increased markedly since 2016 (e.g., [23,24,26,27,28,29,30,31,32,33,34,35,36,37,38] etc.)*.*

The tests were applied to a variety of label types in different jurisdictions, and the outcomes varied, with some reporting significant impacts [39] and others not [26]. An interesting new area has emerged that seeks to quantify the impact of FoP labels on consumer obesity, via their influence on producer reformulation, rather than consumer purchase behavior [17,39,40]. However, producer motivation in this area is likely to be linked to their perception or knowledge of FoP impact upon consumer choice over the longer term. While this increase in FoP testing research is welcome, it has a major shortcoming in that, in most cases, the sample is aware of the purpose of the research [41,42]. This awareness reduces the reliability of the findings, as the prompt that is delivered is likely to significantly impact upon consumer response, and further, does not occur in a real purchase situation.

The second editorial [17] noted this shortcoming, and called for more “real world” (Picture. 8) research to be undertaken using unprompted samples and/or actual purchase data in order to create more reliable performance measures that could justify the deployment or continuation of these labels. In response to Lachat & Tseng’s call for more FoP label performance testing research, the authors conducted research that measured the unprompted impact of the Australasian Health Star Rating (HSR) FoP label system on the choice behavior of a large sample of supermarket shoppers in New Zealand [41]. The researchers conducted the study in late 2014, very shortly after the HSR label’s introduction to New Zealand.

This article reports a replication and expansion of the work to create a longitudinal study of the impact of the HSR system as established in the New Zealand market, and becoming more familiar to consumers. The study thus contributes to the small but growing resource of published tests of unprompted consumer response to FoP label systems. As it uses the unprompted response of consumers in the field, it also accords with Kanter, Vanderlee & Vandevijveres’ (2018) [17] suggestions for more real world research.

### Research Background

**Figure 2:**
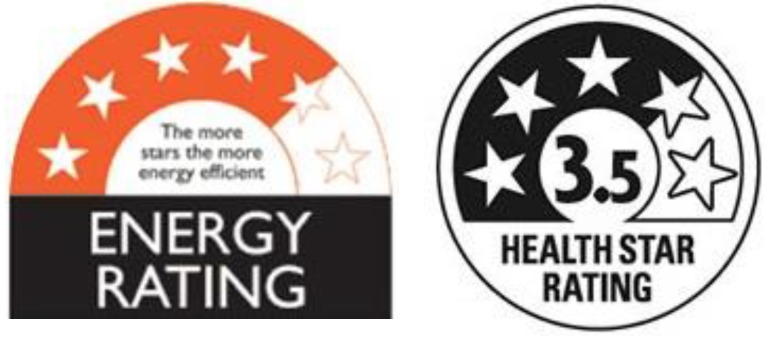
E3 (appliance energy use) and HSR (food nutritional value) product ratings labels.

The HSR FoP nutritional rating system was developed by the Australian New Zealand Food Standards Authority, and was deployed across Australasia on a voluntary basis in 2014 [41,43]. The label system, and its dual-national administration, has clear evolutionary links to the ‘E3′ energy star ratings label system that has been compulsorily applied to large domestic appliances across Australasia for more than 30 years [44] (Figure 2).

The use of a system that was already familiar to Australasian consumers, and had a record of proven performance [45], seemed logical. However, the deployment of another ‘New to the World’ FoP nutritional label system was controversial, and many favored the use of the existing multiple traffic light system that had been developed and heavily researched in the UK [43].

There was also an uncertainty with regard to transferring the consumer durable E3 label format to food products that related to the nature of dual process consumer decision-making [46]. Consumer durable evaluation and purchases are typically associated with ‘slow’, fully reasoned, consumer decision processes, whereas food evaluation and purchases are predominantly characterized by ‘fast’, heuristic-based decisions that are fundamentally different in their inputs and structure [47,48,49]. As a system that works in one consumer decision environment may not necessarily work in another, there was reason to doubt the efficacy of the HSR as an input to food evaluation and choice. This situation was aggravated by the fact that no testing of the impact of the HSR label on unprompted food consumer choice occurred before the HSR was deployed across Australasia [41].

Further issues with the HSR were created by the initial ‘style guide’ for the label [50]. This document allowed a wide range of label formats to be employed, which ranged from fully evaluative to evaluative reductive hybrids, in an almost unlimited variety of sizes, shapes and layouts. Furthermore, unlike the E3 label, no color palette was specified for the HSR. This looseness in specification is highly unusual and undesirable in standard commercial branding practice [51,52]. This, coupled with the fact that the HSR was a voluntary system, allowed a manufacturer to selectively display a very wide range of information in a very wide range of formats to the consumer—or not to display it at all, as they saw fit.

**Figure 3:**
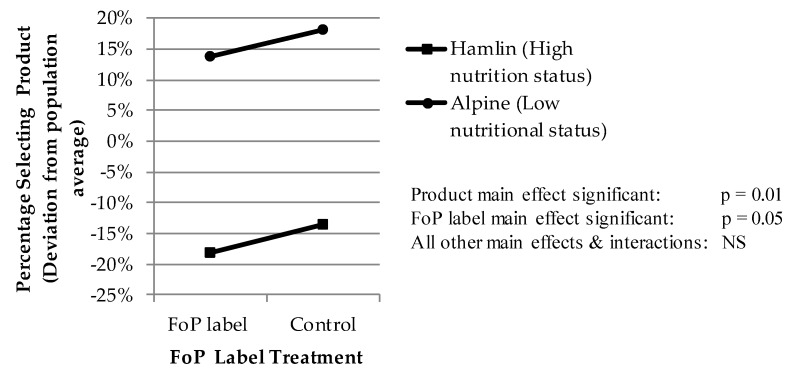
Result of the 2014 trial (Hamlin & McNeill, 2016 [41]).

Hamlin and McNeill (2016) [41] conducted their first test of the HSR in late 2014, within weeks of the introduction of the HSR into the Australasian market. They reported the methodology and outcomes of this test in an article in ‘Nutrients’. The outcome of the test was that the 1200 consumer exit samples both noticed and processed the HSR FoP without any prompting, but that the sample processed the cue as a ‘nominal’ single-state cue. The impact of the FoP label cue was consistently positive, regardless of the number of stars. The conclusion drawn from this result was that the sample processed the HSR FoP just like any other nominal brand cue, which rendered it completely ineffective as a healthy choice moderator, as this response does not allow the consumer to differentiate between high and low nutritional status food products (Figure 3).

The result above was unsurprising, given that the food industry has trained the Australasian food consuming public to process package cues in exactly this ‘nominal’ manner for over a century, and that 99% of all cues, including all major brands that are presented to the food consumer at the retail point of sale, are single-state and nominal in nature. However, it could be reasonably argued that this test was ‘unfair’, in that the HSR label had not been present within the market for long enough for consumers to become properly aware of it, and to learn to recognize and process it as its designers intended. The Authors shared this view, and thus planned at the time, to replicate the research in late 2016.

**Figure 4:**
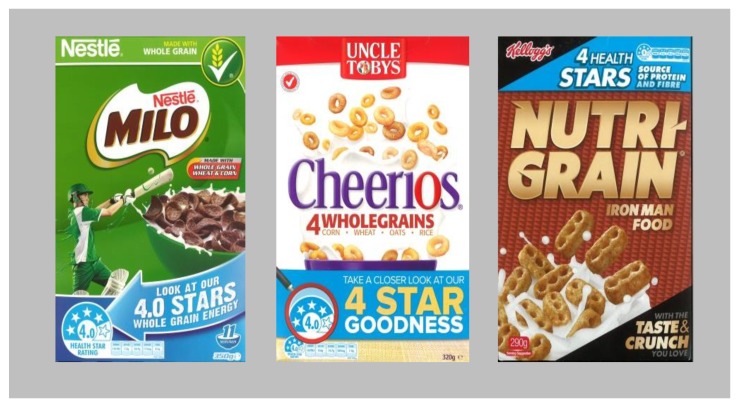
HSR label based ‘superFoP’ cold cereal prime facing designs (New Zealand 2014–2016).

In the intervening period a series of fortuitous events occurred. The research was conducted within the cold breakfast cereal category because this was one of the few categories within a supermarket that already contained high and low nutritional status products that were otherwise closely comparable in their form, price and presentation. The cold breakfast cereal category in New Zealand is dominated by four major producers: Kellogg’s, Uncle Toby’s, Sanitarium and Nestle. For reasons that the authors have not been able to ascertain, a marketing war erupted between these companies immediately after the first research exercise was completed. The war was directly based upon the nutritional status of products as expressed by the HSR. The major players promoted high (four-star plus) HSR scoring products within the category on all national media, including TV, print and the Internet. The packages were also redesigned to incorporate the HSR label, with supporting comments in the form of ‘SuperFoPs’, that occupied up to 25% of the prime facing of the package (rather than the one to two percent which is more usually devoted to FoP label cues) (Figure 4).

This marketing war continued at a moderate to high intensity for the entire 2-year period between the two research exercises. It was also supported by some government communication. Such was the level and nature of targeted marketing expenditure that the authors are confident that no nutritional FoP has ever received such heavy and consistent support from major industry players and government over such a prolonged period in any category and in any jurisdiction. Thus, a combination of design and serendipity established an ideal test environment for the second research exercise.

## 2. Materials and Methods

This research tested the same two research hypotheses as the earlier research:

*H*^1^—The HSR FoP label would significantly influence consumer choice.

*H*^2^—The HSR FoP labels’ impact upon consumer choice would be moderated by variations in the 1–5 ‘star’ rating expressed by the label.

**Figure 5:**
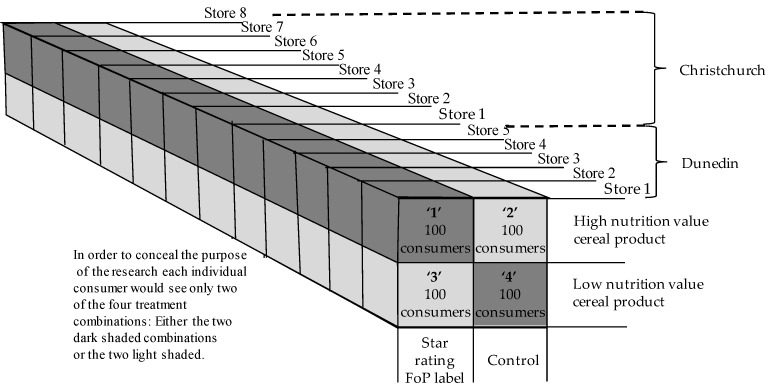
Experimental design.

The replicated 2 × 2 factorial experimental design from the earlier research was used again, with two product treatment levels: one product with a high and one product with a low nutritional status, and two HSR FoP treatment levels: FoP reflecting nutritional status present, and FoP absent (Figure 5).

Two minor modifications were made to the method used in the earlier work: Firstly, rather than six replications completed in one urban center in the far south of New Zealand (Dunedin), 13 replications were completed in two centers: five in Dunedin (pop. 120,000) and eight in Christchurch (pop. 500.000, 350 km to the North of Dunedin).

**Figure 6:**
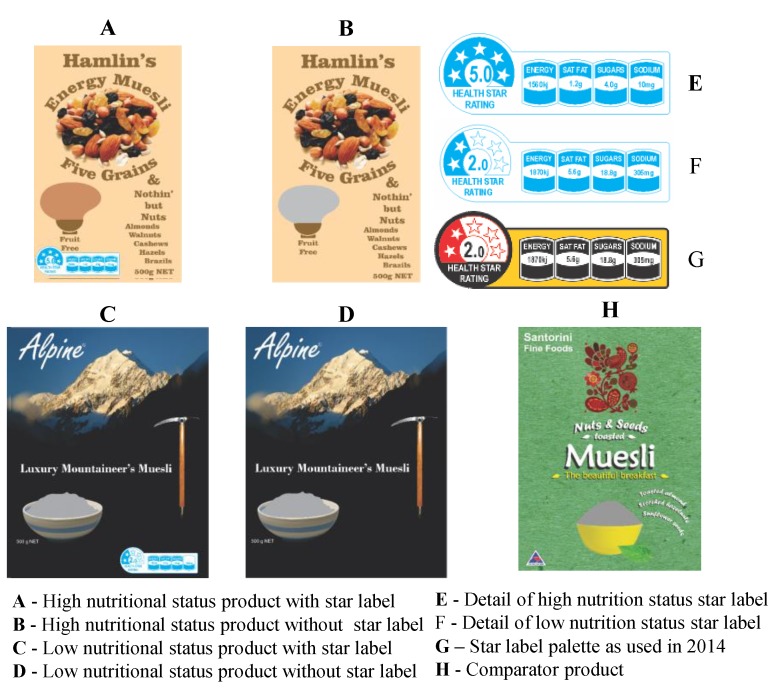
Experimental design—details of products and treatments.

Secondly, the two levels of the HSR FoP label treatment were changed slightly from the earlier work. In the first study, a standard color palate for the HSR label had not been established by ANZFA, so the red, yellow and black palette for the existing E3 electrical appliance rating system was used instead. By 2016, a white and Pantone^®^ ‘Process Blue’ palette had been universally established by the major industry players within the category as the standard for the HSR label (See superFoPs in Figure 4), so the colors of the FoP label treatments were altered to reflect this development (Figure 6).

The two levels of product that carried the FoP labels remained unchanged from the earlier work and were: a cold ready to eat muesli product with high (five star) nutritional status (level 1) and an equivalent product with low (two star) nutritional status (level 2). If the FoP star rating label was present on a product, then it was adjusted to exactly reflect the nutritional status of the product to which it was applied [41]. The HSR style guide did not specify any size, so the mark was set to 2.7% of the prime face of each product in the earlier work. This percentage is consistent with current practice within the category worldwide when the HSR is not presented as a superFoP, so it was left unchanged.

The four treatment combinations were presented to the consumer sample as high quality, complete product mock-ups. The front facings of these four products and of the ‘comparator’ product that represented the choice alternative in the research are shown in Figure 6.

**Figure 7:**
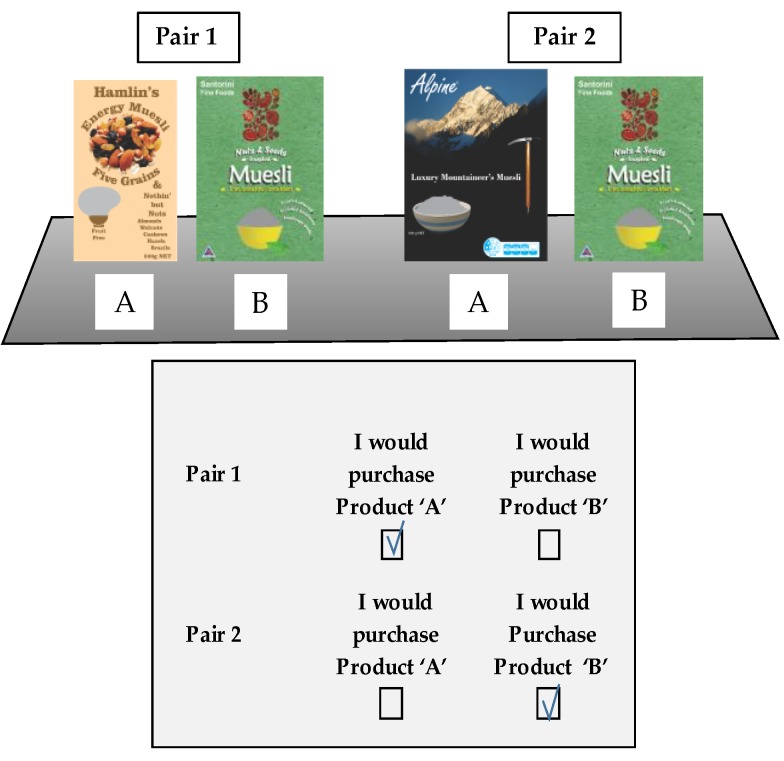
Experimental design, presentation of FoP labels/products and instrument.

The dependent variable in this study is consumer choice. Each consumer was presented with two (of the four) treatment combinations, arranged as two pairs placed next to a common comparator product (Figure 7). The consumer was asked to indicate which of the two products they would choose by circling either ‘A’ (comparator) or ‘B’ (treatment) on their response form (Figure 7). Each treatment combination was evaluated by 100 consumers.

The derived dependent variable used in the analysis was the percentage of the 100 consumers that selected the treatment combination over the comparator. The large sample allowed this number to be treated as a parametric input to an analysis of variance. The analysis of variance was a simple two-way analysis of variance with replications undertaken on Excel.

The consumers/participants were recruited by qualified intercept (purchased breakfast cereals by trolley observation) on exit from the supermarkets belonging to the same chain (New World). As the research only took less than one minute in each case, it could be fully administered at the store’s exit. The factorial design allowed each consumer to evaluate two treatment/comparator combinations without the purpose of the research becoming apparent to them, so that 200 consumers were required for each 2 × 2 factorial exercise with 100 observations in each of the four cells. As the consumers were unaware of the purpose of the research, their response to the decision tasks were therefore unprompted. A small bar of chocolate was given to each consumer as a courtesy at the conclusion of their participation. The exercise received ethical approval from the University of Otago Ethics Committee, and participant consent was sought at each intercept. Participants were able to withdraw from the exercise at any stage.

The exercises in Dunedin were conducted on a Friday and Saturday on three successive weeks in October 2016, generating a total consumer sample size of 1000 individuals and 2000 evaluations. The research in Christchurch was conducted in a similar manner over 4 weeks in November with 1600 consumers and 3200 observations.

## 3. Results

The results of the research in Dunedin and Christchurch were treated as separate exercises because urban communities in New Zealand are quite isolated from one another and each as a result has its own culture (see Supplementary file). The researchers, therefore, decided to treat this potentially large source of variation as an inter-study replication, to be dealt with by subjective analysis, rather than as an intra-study replication to be addressed by statistical analysis—as the individual stores in each center were treated.

**Figure 8:**
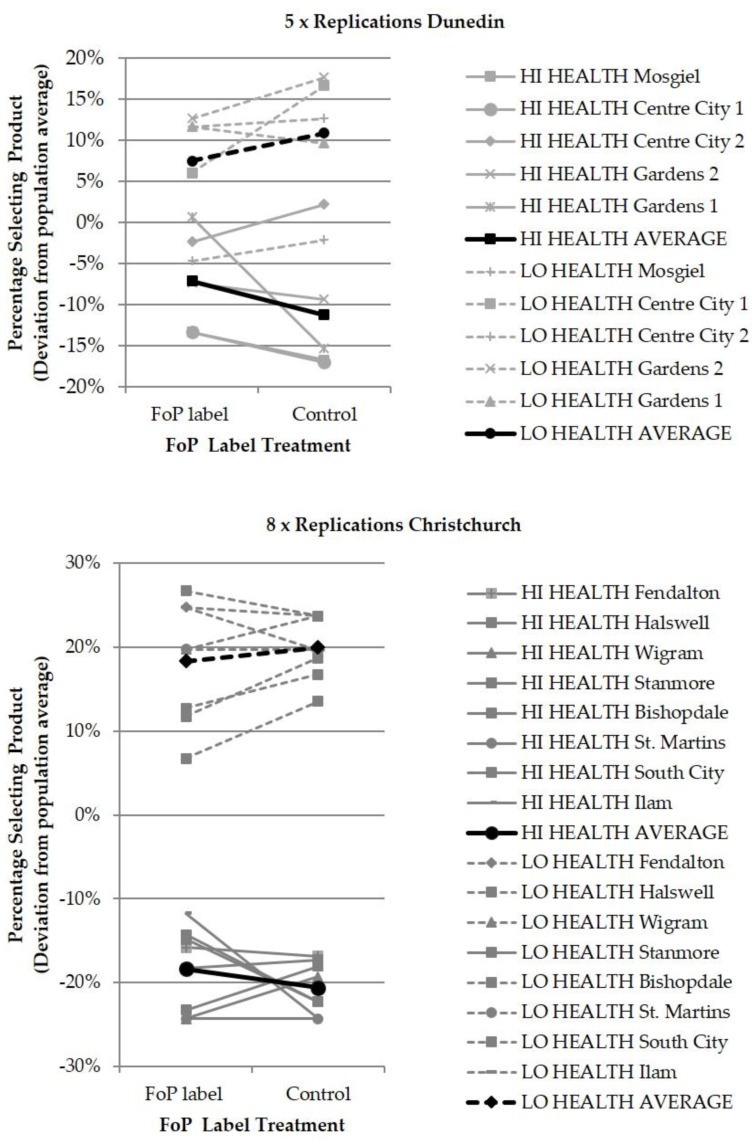
Results graphical presentation.

The results for each center are presented graphically in Figure 8, and the analyses of variance are shown in Table 1, Table 2, Table 3 and Table 4. Two analyses of variance tables are presented for each center. The first table presents an analysis of variance in which the research exercises at each store are treated as replications, and the second table presents the same data in which the stores are treated as independent variables in their own right and all potential main effects and interactions between the package FoP and store are considered. The two analyses show that the outcome with regard to *H*^1^ and *H*^2^ is stable/consistent between the two centers, and is also robust to the method of analysis of variance that is applied to them. The graphical results in Figure 8 showing the detail variations in outcome between individual stores/replications also demonstrate why multiple replications of a significant size are required to generate a reliable result.

*H*^1^—The HSR FoP label would significantly influence consumer choice.

The two analyses show that there is only a single observable significant main effect of the HSR FoP; a weak/borderline *p* < 0.1 significance emerges in Christchurch if stores are treated as an independent variable. The result indicates a weak and general positive impact of both label treatments (two star & five star) on consumer preference, with the statistical significance depending on the statistical analysis used. *H*^1^ is therefore not strongly supported.

This outcome differs from that of the first study in 2014 which showed a general positive impact on consumer preference that was substantial, positive and identical for each of the two HSR label treatments. The level of the statistical significance *p* < 0.05 in the 2014 study was also not sensitive to the statistical analysis used.”

*H*^2^—The HSR FoP labels’ impact upon consumer choice would be moderated by variations in the 1–5 ‘star’ rating expressed by the label.

The two analyses show a consistent outcome for *H*^2^. The graphical representations of the consolidated results for each center (black line) are presented in Figure 8. These show that the two lines are not parallel, but in fact show a convergence that is consistent with the five star HSR nutrition label increasing demand for the high nutritional value product and the two star HSR label suppressing demand for the low nutritional value product. However, in all four analysis of variance tables, the effect is not statistically significant—Thus *H*^2^ is not supported either.

The effect of the FoP label is not consistent replication to replication, and is much smaller than the effect of the products themselves, which are consistently *well separated* in all replications. It must also be noted that the products themselves were designed to be neutral in their impact, and contained no strong brand cues or brand-related design features. The same applies to the stores/replications, which were as similar to each other as recruitment would allow, but still developed data variations that exceeded the impact of the HSR labels in both exercises. Thus, the impacts of large variations in the state of the HSR FoP were less significant than those of two other independent (extraneous) variables that were themselves designed to be insignificant, and the statistical outcome of non-significance of the HSR FoP is thus consistent with observation of Figure 8.

## 4. Discussion and Conclusions

This research exercise has delivered a robust result that contains both positive and negative outcomes relating to the implementation of the Australasian Health Star Rating FoP. The results of the 2014 research exercise indicated that the consumer sample attended to and processed the HSR, but that they did so in a manner that was consistent with any other nominal, commercial brand. The 2014 sample processed the HSR as an icon with positive connotations, regardless of what differing levels of nutritional status the HSR displayed—thus rendering the HSR completely ineffective in its planned role as a moderator of behavior based on nutritional value. i.e., the appearance on pack of the HSR alone was enough for consumers to view the product in a positive nutritional light.

In 2016, this nominal cue processing effect had been virtually eliminated, and a visible, differential effect of the desired ordinal cue type can now be observed. However, this differential effect remains statistically insignificant and tiny compared to even minor variations in the purchase environment and cue vehicle. Thus, it can be concluded that despite the provision of massive national marketing support in all media over 2 years, the HSR remains ineffective in influencing unprompted consumer choice between food products within the category selected for study.

This research and its conclusions do have limitations. The study was restricted to a single category, within a single retail platform and in a single country, placing limits on the generalizability of these conclusions. However, the chosen category has one of the widest nutritional status spreads of any category, and has a heavy penetration of HSR application in what is still a voluntary environment.

Also, no other category environment worldwide has had the same concentration of marketing support for an FoP delivered to it over this period. Thus, if the HSR FoP was going to deliver a positive result anywhere, it would have done so here. While this is not a result that many backers of the HSR would welcome, the observed consumer response to this system does appear to be moving in the right direction, and certainly to a degree that justifies another longitudinal replication in 2018/9 to see if further progress in that direction has been made.

On a final methodological note, the authors would like to draw attention to the fact that the solidity of this research result and its predecessor is based upon extensive intra-study replication of a type that is becoming increasingly unfashionable in many areas of human research, as expressed by this recent commentary in a leading marketing journal:

“…. we take the position that intra-study replication does not represent improvement in replication in marketing research as the approach reflects an inaccurate assessment of true replication practice and thus misrepresents reality. In marketing, as in other fields, replication by independent researchers is required to advance science and represent the true reality of phenomena.” We do not mean to suggest that intra-study replication has no place in marketing research, only that it is mistake to elevate the approach to same level as the inter-study replication.”

([53], p. 267).

These results also quite clearly demonstrate that intra-study replication is in fact essential to achieve a reliable result upon which any inter-study replication might be based. The one form of replication is in fact a pre-requisite for the other—a fact that has been well understood within Agricultural Science since the 1920s [54]. In this case, the store replications can be considered to be an intra-study replication exercise, while the two centers are inter-study replications.

Each one of the replications in either center could have been published as a stand-alone study—there are many examples of published research that bases conclusions on single exercises that use smaller samples. A single glance at Figure 8 clearly indicates what the potential variations in the outcomes of such single sample exercises might be, and what the implications of reporting them separately would be for the long-term integrity of the research literature.

## Figures and Tables

**Table 1:** Analysis of variance Dunedin, stores as replications.

	SS	DF	MS	F	Significance
Total	0.2652	19			
Packages	0.1689	1	0.17	30.3	***
FoP label	0.0001	1	0.00	0.0	NS
Package × FoP	0.0071	1	0.01	1.3	NS
Error	0.0891	16	0.01		

SS—Sum of Squares; DF—Degrees of Freedom; MS—Mean Square; F—‘F’ ratio; ***—Significant at *p* < 0.01; NS—Not significant.

**Table 2:** Analysis of variance Dunedin, stores as third independent variable.

	SS	DF	MS	F	Significance
Total	2.4480	59			
Pack	2.1462	1	2.1462	556.0	***
FoP	0.0090	1	0.0090	2.3	NS
Store	0.0502	4	0.0126	3.3	*
Pack × FoP	0.0001	1	0.0001	0.0	NS
Pack × Store	0.0486	4	0.0121	3.1	*
Stores × FoP	0.0244	4	0.0061	1.6	NS
Pack × FoP × Store	0.0151	4	0.0038	1.0	NS
Error	0.1544	40	0.0039		

SS—Sum of Squares; DF—Degrees of Freedom; MS—Mean Square; F—‘F’ ratio; ***—Significant at *p* < 0.01; *—Significant at *p* < 0.10; NS—Not significant.

**Table 3:** Analysis of variance Christchurch, stores as replications.

	SS	DF	MS	F	Significance
Total	1.2647	31			
Packages	1.1915	1	1.19	474.8	***
FoP label	0.0001	1	0.00	0.0	NS
Package × FoP	0.0029	1	0.00	1.2	NS
Error	0.0703	28	0.00		

SS—Sum of Squares; DF—Degrees of Freedom; MS—Mean Square; F—‘F’ ratio; ***—Significant at *p* < 0.01; NS—Not significant.

**Table 4:** Analysis of variance Christchurch, stores as third independent variable.

	SS	DF	MS	F	Significance
Total	2.4480	95			
Pack	2.1462	1	2.1462	889.6	***
FoP	0.0090	1	0.0090	3.7	*
Store	0.0502	7	0.0072	3.0	*
Pack × FoP	0.0001	1	0.0001	0.0	NS
Pack × Store	0.0486	7	0.0069	2.9	*
Stores × FoP	0.0244	7	0.0035	1.4	NS
Pack × FoP × Store	0.0151	7	0.0022	0.9	NS
Error	0.1544	64	0.0024		

SS—Sum of Squares; DF—Degrees of Freedom; MS—Mean Square; F—‘F’ ratio; ***—Significant at *p* < 0.01; *—Significant at *p* < 0.10; NS—Not significant.

## Supplementary Materials

The following are available online at http://www.mdpi.com/2072-6643/10/7/906/s1, File S1: Hamlin McNeill 2018 data.

## Abbreviations

ANZFA	Australia New Zealand Food Standards Authority
FoP	Front of Pack
HSR	Health Star Rating
NIP	Nutrition Information Panel
TLL	Traffic Light Label
UK	United Kingdom
